# DMBT1 is upregulated in cystic fibrosis, affects ciliary motility, and is reduced by acetylcysteine

**DOI:** 10.1186/s40348-022-00136-0

**Published:** 2022-03-05

**Authors:** Alexander Kiefer, Erika Plattner, Renate Ruppel, Christel Weiss, Zhe Zhou-Suckow, Marcus Mall, Marcus Renner, Hanna Müller

**Affiliations:** 1 Department of Pediatrics, University Hospital Erlangen, University of Erlangen-Nürnberg, Loschgestr. 15, 91054 Erlangen, Germany; 2 Department of Pediatric Pneumology and Allergology, St. Hedwig’s Hospital of the Order of St. John, University Children’s Hospital Regensburg (KUNO), Steinmetzstr. 1-3, 93049 Regensburg, Germany; 3 Department of Medical Statistics and Biomathematics, University Hospital Mannheim, Theodor-Kutzer-Ufer 1-3, 68167 Mannheim, Germany; 4 Department of Translational Pulmonology, Translational Lung Research Center Heidelberg (TLRC), German Center for Lung Research (DZL), University of Heidelberg, Im Neuenheimer Feld, 69120 Heidelberg, Germany; 5 Department of Pediatric Pulmonology, Immunology and Critical Care Medicine, Charité-Universitätsmedizin Berlin, Augustenburger Platz 1, 13353 Berlin, Germany; 6 Institute of Pathology, University of Heidelberg, Im Neuenheimer Feld 224, 69120 Heidelberg, Germany; 7 Department of Pediatrics, Neonatology and Pediatric Intensive Care, University of Marburg, Baldingerstraße, 35043 Marburg, Germany; 8 Department of Pediatrics, Division of Neonatology and Pediatric Intensive Care, University Hospital Erlangen, University of Erlangen-Nürnberg, Loschgestr. 15, 91054 Erlangen, Germany

**Keywords:** Cystic fibrosis, DMBT1, Inflammation, Ciliary motility, Acetylcysteine

## Abstract

**Background:**

Cystic fibrosis (CF) is the most common genetic disorder in the Caucasian population. Despite remarkable improvements in morbidity and mortality during the last decades, the disease still limits survival and reduces quality of life of affected patients. Moreover, CF still represents substantial economic burden for healthcare systems. Inflammation and infection already start in early life and play important roles in pulmonary impairment. The aim of this study is to analyze the potential role of DMBT1, a protein with functions in inflammation, angiogenesis, and epithelial differentiation, in CF.

**Results:**

Immunohistochemically DMBT1 protein expression was upregulated in lung tissues of CF patients compared to healthy controls. Additionally, pulmonary expression of *Dmbt1* was approximately 6-fold increased in an established transgenic mouse model of CF-like lung disease (ENaC tg) compared to wild-type mice as detected by qRT-PCR. Since acetylcysteine (ACC) has been shown to reduce inflammatory markers in the airways, its potential influence on DMBT1 expression was analyzed. A549 cells stably transfected with an expression plasmid encoding the largest (8kb) DMBT1 variant (DMBT1+ cells) or an empty vector control (DMBT1- cells) and incubated with ACC both showed significantly reduced DMBT1 concentrations in the culture medium (*p* = 0.0001). To further elucidate the function of DMBT1 in pulmonary airways, respiratory epithelial cells were examined by phase contrast microscopy. Addition of human recombinant DMBT1 resulted in altered cilia motility and irregular beat waves (*p* < 0.0001) suggesting a potential effect of DMBT1 on airway clearance.

**Conclusions:**

DMBT1 is part of inflammatory processes in CF and may be used as a potential biomarker for CF lung disease and a potential tool to monitor CF progression. Furthermore, DMBT1 has a negative effect on ciliary motility thereby possibly compromising airway clearance. Application of ACC, leading to reduced DMBT1 concentrations, could be a potential therapeutic option for CF patients.

**Supplementary Information:**

The online version contains supplementary material available at 10.1186/s40348-022-00136-0.

## Introduction

Since the first description of cystic fibrosis (CF) in 1938 the median age of survival has increased progressively, reaching over 40 years in developed countries [1]. Progression of CF is characterized by inflammation, viral, and bacterial infection, and deterioration of lung function due to structural changes [2]. Accordingly, pulmonary tissues in advanced stages show pronounced inflammation resulting in increased pulmonary symptoms and decreased lung function [3]. Furthermore, infections with distinct bacteria, e.g., Staphylococcus aureus and Pseudomonas aeruginosa as well as general pulmonary bacterial load contribute to airway inflammation and lung damage [4]. Therefore, many efforts have been made to reduce inflammation and bacterial colonization in children with CF. Previous research has used mice with airway-specific overexpression of the β-subunit of the epithelial Na+ channel (βENaC; encoded by the *Scnn1b* gene) showing a CF-like phenotype with increased absorption of Na+ and fluid from the airway lumen, increased mucus concentration, delayed mucus transport and mucus adhesion. In analogy to humans with CF, these mice suffer from airway surface dehydration leading to mucus obstruction, chronic inflammation, reduced bacterial clearance, and emphysema [5–8]. Airway inflammation is marked by transient perinatal recruitment of macrophages and eosinophils and expression of tumor necrosis factor-alpha and IL-13 whereas persistent increases in neutrophils, keratinocyte-derived cytokine, and chitinases have been described [7].

Another protein with known functions in innate immunity and inflammation and therefore a possible role in dysregulated inflammation during CF is named DMBT1 (deleted in malignant brain tumors 1; alternative names: glycoprotein-340 (gp-340) or salivary agglutinin (SAG)). DMBT1 is a secreted scavenger receptor cysteine-rich (SRCR) protein, of the adult respiratory tract expressed in alveolar type II cells, epithelial cells and associated glands. Steady-state DMBT1 levels appear to be low to moderate in the lung of healthy adults but increased during inflammation and bacterial and viral infection [9, 10]. Moreover, the percentage of DMBT1-positive type II pneumocytes has been shown to positively correlate with severity of inflammation. In infants pulmonary DMBT1 expression has likewise been observed in epithelial cells and associated glands [11, 12]. In analogy to adults, DMBT1 expression is upregulated during inflammatory processes in the newborn lung, for example respiratory distress syndrome where DMBT1 is detected in hyaline membranes and inactivates different surfactant preparations in a dose-dependent manner [11, 12]. Multiple mechanisms of action of DMBT1 have been described: DMBT1 binds to other proteins with functions in innate immunity (e.g., secretory IgA, surfactant protein D, surfactant protein A) [13], aggregates diverse species of pathogenic bacteria using the VEVLXXXXW motif in its scavenger receptor cysteine-rich domains or glycosylation [14, 15], functions as a pattern-recognition molecule for poly-sulfated and poly-phosphorylated ligands. These findings provide a molecular basis for its broad bacterial-binding specificity and inhibitory effects on lipopolysaccharide-induced Toll-like receptor 4 (TLR4)-mediated nuclear factor kappa B activation [16, 17]. DMBT1 binds also various viruses (e.g., influenza A virus and human immunodeficiency virus type I, hepatitis B) [13, 18]. Therefore, DMBT1 is found in the respiratory tract as well in the gastrointestinal tract and in lacrimal fluid to be part of innate immunity and to be part of regulations against inflammation [18]. DMBT1 has already been examined in several inflammatory diseases like Crohn's disease, active bacteria-related appendicitis and bacterial endocarditis [17, 19, 20]. Besides inflammation, DMBT1 has functions in angiogenesis and epithelial differentiation illustrating characteristics with important impact in tissue repair [13, 21].

The aim of this study is to analyze the potential role of DMBT1 in CF. We examined pulmonary DMBT1 expression in patients with CF, the effect of DMBT1 on motility of ciliated respiratory epithelium and the potential therapeutic use of acetylcysteine (ACC) to reduce DMBT1 levels and hence inflammation.

## Methods

### Immunohistochemistry

Formalin-fixed and paraffin-embedded lung sections of patients with CF were analyzed by immunohistochemistry to detect DMBT1. Post-mortem examinations of patients with CF are very rare since the diagnosis is normally made during lifetime. Therefore, post-mortem lung sections of only one case of CF were available for staining. Additionally, lung tissue of 13 patients with CF (age: 29.26 ± 1.9 years) who had undergone lung transplantation was stained. Two corresponding persons without lung disease were included as control subjects. The study was approved by the responsible ethics committees of the University of Heidelberg (No. 361/2003) and the University of Erlangen-Nürnberg (No. 443_19B). DMBT1 protein expression was detected using the polyclonal antibody anti-DMBT1p84 and a protocol described earlier [11, 12, 22].

### Cell culture and incubation with ACC

Human lung epithelial A549 cells showing the characteristic morphology of type II lung epithelial cells with typical lamellar bodies were used to examine the effect of ACC on DMBT1 expression [22–24]. Briefly, the cells were stably transfected with an expression plasmid encoding the largest (8 kb) DMBT1 variant (DMBT1+ cells) under the control of a constitutive promoter or an empty vector control (DMBT1− cells) [16]. The cells were cultured in DMEM/F-12 (1:1) + GlutaMAX TM medium (Gibco, Thermo Fisher, Karlsruhe, Germany) supplemented with 10% fetal bovine serum (PAN Biotech, Aidenbach, Germany) and 1% penicillin/streptomycin (Sigma-Aldrich Life Science, Taufkirchen, Germany). Hygromycin B (Carl Roth GmbH + Co. KG, Karlsruhe, Germany; final concentration: 500 μg/ml) was added to keep selection pressure on the cells with inserted plasmids. The DMBT1− and DMBT1+ cells were seeded in 6-well plates. After reaching 90–100% confluence, medium was removed and wells were washed with PBS and incubated in medium without fetal bovine serum for 2 h. Then, ACC (Hexal AG, Holzkirchen, Germany; final concentration: 15 mM) or sodium chloride 0.9% as control was added for another 2 h. The medium was collected and frozen at – 80 °C until determination of DMBT1 concentration by ELISA. Subsequently, the cells were washed again with PBS and medium without fetal bovine serum was added onto the cell layer. After 24 h, medium was removed and frozen for DMBT1 determination.

### Quantification of DMBT1 in supernatants of DMBT1− and DMBT1+ A549 cells by ELISA

The DMBT1 concentrations in the supernatants of DMBT1− and DMBT1+ cells were determined by ELISA (Abbexa, Cambridge, UK) according to the manufacturer’s instructions. The ELISA measured DMBT1 concentrations in the range of 0.156 ng/ml to 10 ng/ml. The sensitivity of the ELISA was < 0.055 ng/ml. Supplemental Figure 1 demonstrated the standard curve of the ELISA. The DMBT1 concentrations of A549 cells incubated with sodium chloride 0.9% instead of ACC were assigned as 100% (control).

### Motility of ciliated respiratory epithelium

Two nasal swabs with respiratory epithelial cells were collected from each of 10 healthy volunteers (6 males, 4 females) after giving informed consent. The swabs were rotated in a tube with 2 ml of cell culture medium (RPMI 1640, HEPES, Thermo Fisher Scientific, Schwerte, Germany) to separate the respiratory epithelial cells from the swab. Human recombinant DMBT1 (hrDMBT1; final concentration: 0.5 μg/ml) was added to one sample and PBS as control to the second sample of each volunteer. Phase contrast microscopy was used to investigate the motility of the cilia.

### Animals

All animal studies were approved by the Regierungspräsidium Karlsruhe, Germany. The generation of ENaC-transgenic mice (line 6608) has been previously described [6]. The colony was maintained on a mixed genetic background (C57BL/6N × C3H/HeN), and ENaC-transgenic mice were identified by PCR as described [6, 7]. Wild-type littermates served as control animals. Mice were housed in a specific pathogen-free animal facility and had free access to chow and water. At the age of 6 weeks, mice were sacrificed and lungs were stored as samples to isolate RNA for later expression studies (Applied Biosystems, Darmstadt, Germany).

### Quantitative RT-PCR analyses

Single-stranded cDNA synthesis was done with 300 ng of total RNA and oligo-dT primers according to standard procedures. Quantitative RT-PCR experiments were carried out using 5 ng of reverse transcribed RNA per reaction and TaqMan assays-on-demand (Thermo Fisher Scientific, Karlsruhe, Germany) according to the manufacturer’s instructions. All PCR reactions were done in triplicate. Signal detection was performed with the ABI Prism 7900HT Sequence detection system (Thermo Fisher Scientific, Karlsruhe, Germany). Cycle threshold (Ct) values were normalized against Ct values obtained for mouse beta-actin (Mm00607939_s1). All primer assays used during this study are listed in Table 1.

**Table 1:** Primer assays used for quantitative RT-PCR analysis

Assay ID	Gene symbol	Gene name
Mm00455996_m1	*Dmbt1*	Deleted in malignant brain tumors 1
Mm00499170_m1	*Sftpa*	Surfactant protein A
Mm00486060_m1	*Sftpd*	Surfactant protein D
Mm00436945_m1	*Tff1*	Trefoil factor 1
Mm00447491_m1	*Tff2*	Trefoil factor 2 (spasmolytic protein 1)
Mm00445274_m1	*Tlr4*	Toll-like receptor 4

### Statistics

Statistical analysis was performed with SAS software, release 9.4 (SAS Institute Inc., Cary, NC, USA). To analyze quantitative RT-PCR data, *t* test (Gosset) and Welch-Satterthwaite *t* test was applied. To compare DMBT1 concentrations in cell culture media, paired *t* test was used for pairwise comparisons and *t* test for independent samples was used for unpaired samples. Variance analysis for paired values was used to analyze cilia motility data. In general, test results with *p* values less than 0.05 were regarded as statistically significant.

## Results

### DMBT1 protein expression in pulmonary tissue of patients with CF

**Fig. 1:**
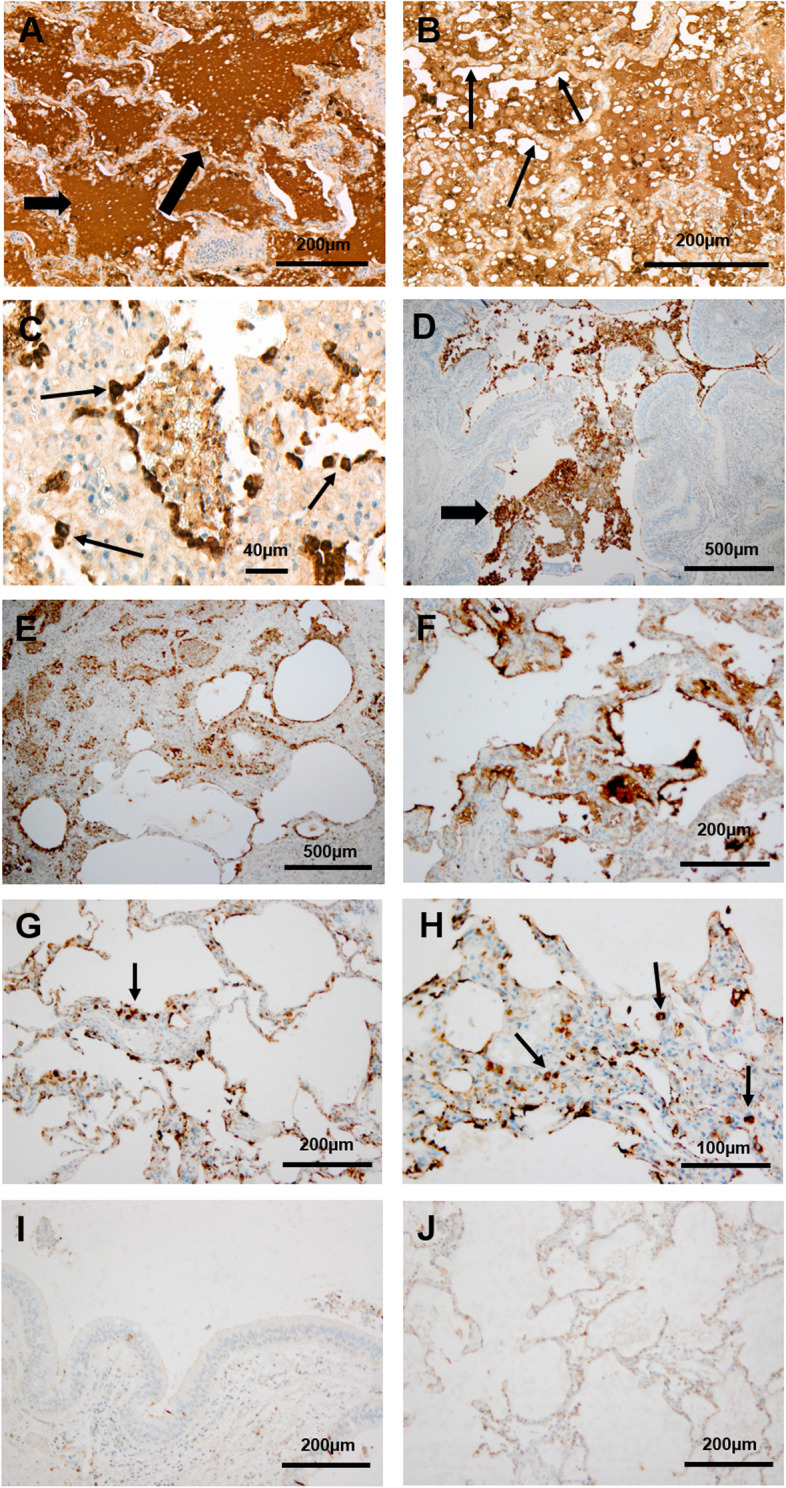
Pulmonary DMBT1 expression in patients with CF and corresponding controls. Immunohistochemical analysis of DMBT1 expression in post-mortem lung tissue of a patient with cystic fibrosis (**A**–**C**), in explanted lungs of CF patients due to lung transplantation (**D**–**H**) and in corresponding controls without lung disease (**I**, **J**). DMBT1 localization is displayed as brown staining. **A** The alveoli and small airways contained luminal mucus with intense DMBT1 staining (arrows). Magnification: × 10. **B** The respiratory epithelial cells (small arrow) showed high expression of DMBT1. Magnification: × 15. **C** Multiple DMBT1-positive macrophages (arrows) were observed in the aveoli and small airways. Magnification: × 40. **D**–**F** The small airways (**D**, arrow) and alveoli (**E**, **F**) showed mucus stained with immunohistochemistry using an antibody against DMBT1. Magnification: × 4. **G**, **H** DMBT1-positive macrophages (arrows) were visible in the aveoli (small arrows). Magnification: × 10 (**G**) and × 20 (**H**). **I**, **J** Control tissue without lung disease showed only distinct DMBT1 expression compared to pulmonary DMBT1-expression in CF patients. Magnification: × 10

The examined lung sections were obtained from a 20-year-old male patient with CF who died due to increasing respiratory insufficiency. Immunohistochemical staining showed highly upregulated DMBT1 protein in respiratory epithelial cells. Additionally, the alveoli and small airways were filled with DMBT1-positive mucus and multiple DMBT1-positive macrophages (Fig. 1A–C). Similar results were seen lung sections from 13 patients who had undergone lung transplantation due to CF (Fig. 1D–H). Lung tissue without lung disease served as control and showed only distinct DMBT1 expression as already described in literature (Fig. 1I, J).

### *Dmbt1* expression in pulmonary tissue of a transgenic mouse model of CF-like lung disease detected by quantitative RT-PCR

**Fig. 2:**
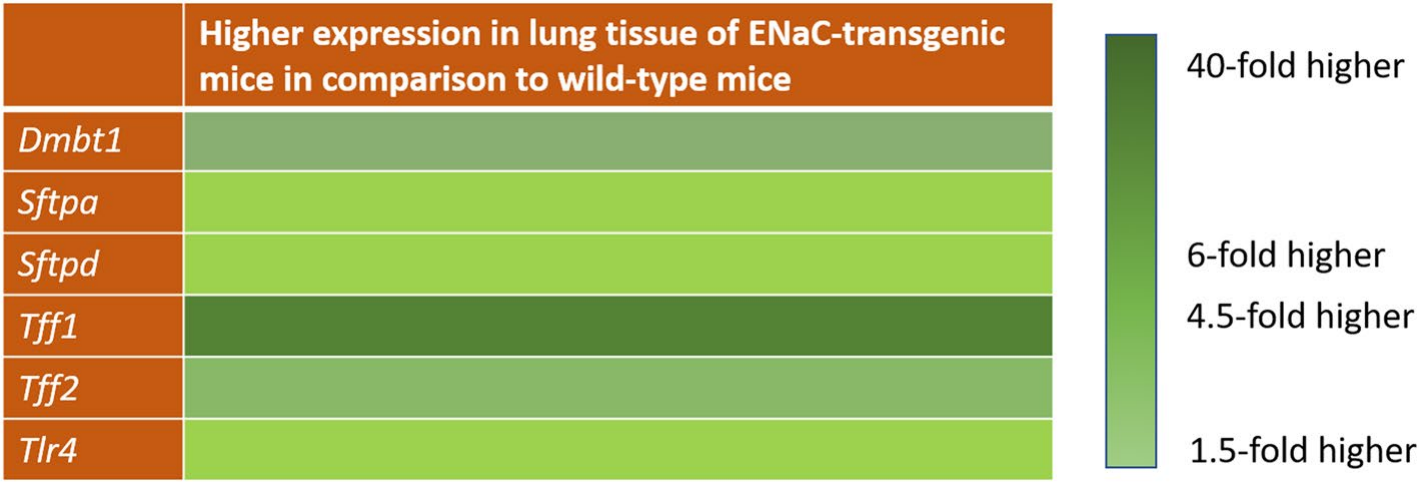
Expression of *Dmbt1* and interaction partners in a transgenic mouse model of cystic fibrosis-like lung disease. *Dmbt1* and its binding partners *surfactant protein A*, *surfactant protein D* and *Trefoil factor 2* were upregulated in the ENaC transgenic mouse model of cystic fibrosis-like lung disease (ENaC-tg mice) compared to wild-type control animals (wt mice) using qRT-PCR. *ß-actin* and *Tfrc* were used as housekeeping genes. Data were from 3 mice per group

To determine *Dmbt1* expression in a mouse model of CF-like lung disease we analyzed the lungs of 3 ENaC-transgene and 3 wild-type mice (Fig. 2). Pulmonary expression of *Dmbt1* was approximately 6-fold increased mice with CF-like lung disease compared to wild-type littermates (*p* = 0.0908). Different known binding partners of Dmbt1, including SftpA, SftpD, and Trefoil factor 2 (Tff2) are also expressed in the lung. Therefore, we analyzed the expression levels of these genes together with the two proinflammatory markers Tlr4 and Tff1 and observed upregulation in the mouse model of CF-like lung disease compared to wild-type mice (Fig. 2): *SftpA* nearly 1.5-fold (*p* = 0.0475), *SftpD* 1.7-fold (*p* = 0.0954), *Tff1* approximately 40-fold (*p* = 0.1547), *Tff2* 4.5-fold (*p* = 0.0482), and *Tlr4* 1.5-fold (*p* = 0.1507). *Trefoil factor 3* was neither detectable in the lungs of wild-type mice nor in those of the CF-like lung disease. The expression level of *Dmbt1* was lower in comparison to that of *SftpA* and *SftpD*.

### Effect of ACC on DMBT1 concentration in the supernatant of DMBT1− and DMBT1+ cells

**Fig. 3:**
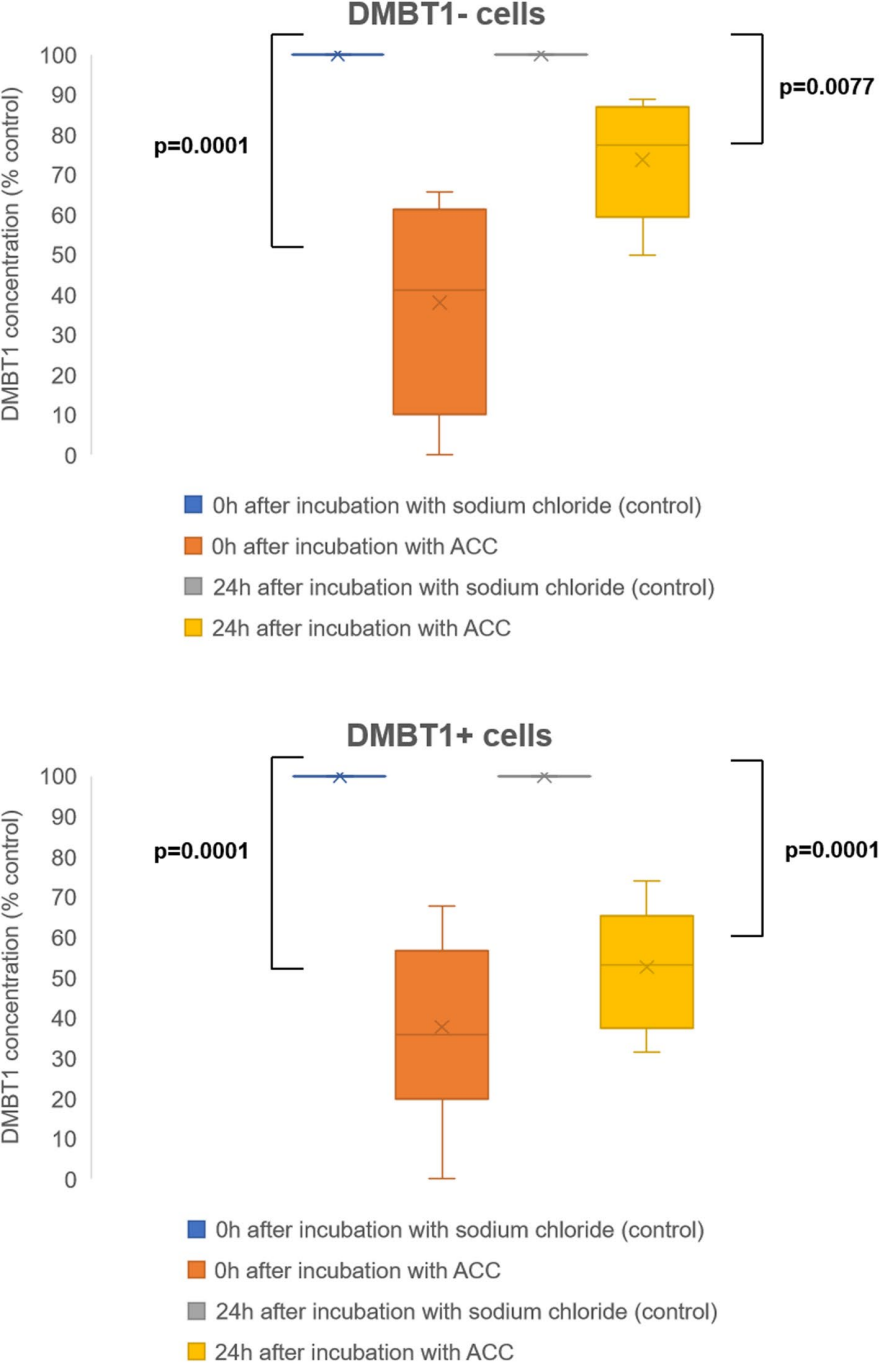
DMBT1 concentration in the supernatant of DMBT1− and DMBT1+ A549 cells is reduced by acetylcysteine. ELISA analysis of DMBT1 production in A549 cells stably transfected with a plasmid encoding the largest DMBT1 isoform or an empty control plasmid. Cells were either treated with 15 mM acetylcysteine (ACC) or sodium chloride as control for 2 h and DMBT1 concentration was measured 0 and 24 h after incubation. Values are expressed as box plots of 3 replicates. ACC: acetylcysteine

Addition of ACC to the cell culture medium led to a significantly reduced DMBT1 concentration in the medium of both, DMBT1− and DMBT1+ cells (both cell types: *p* = 0.0001; Fig. 3). There was no significant difference in the magnitude of DMBT1 reduction between DMBT1− and DMBT1+ cells (*p* = 0.9829). Additionally, a similarly significant effect of DMBT1 reduction was observed 24 h after termination of ACC incubation (DMBT1− cells: *p* = 0.0077; DMBT1+ cells: *p* = 0.0001); however, the DMBT1+ cells showed a more pronounced reduction compared to the DMBT1− cells (*p* = 0.0270) (Fig. 3).

### Effect of human recombinant DMBT1 (hrDMBT1) on ciliary motility of human respiratory epithelial cells

**Fig. 4:**
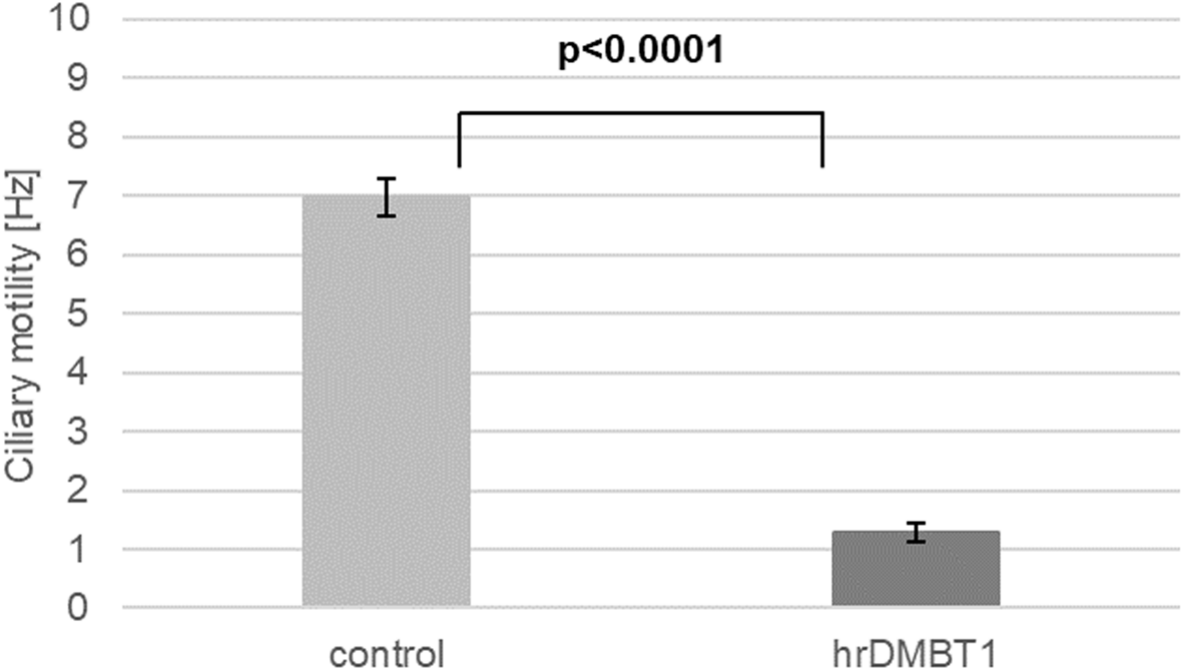
Addition of human recombinant DMBT1 (hrDMBT1) negatively influences cilia motility of human respiratory epithelial cells. Ciliary motility of human epithelial cells treated with human recombinant DMBT1 or phosphate-buffered saline (control) as assessed by phase contrast microscopy. Values are expressed as mean ± SEM

Phase contrast microscopy of untreated control respiratory epithelial cells (Supplemental Video 1: ciliary motility without hrDMBT1) showed a regular motility of the cilia, whereas addition of hrDMBT1 led to significantly altered ciliary motility and irregular beat waves (Supplemental Video 2: ciliary motility with hrDMBT1), suggesting a negative effect of DMBT1 on ciliary motility (*p* < 0.0001; Fig. 4).

## Discussion

### Pulmonary DMBT1 expression and functions in patients with CF

DMBT1 is expressed by alveolar type II cells, epithelial cells, and associated glands in infants and adults [9–12]. The results obtained from human and mouse lung tissue showed that DMBT1 expression was upregulated in CF which goes in line with the known functions of DMBT1 during inflammation. CF is characterized by multiple inflammatory processes; hence, upregulation of DMBT1 seems to be involved in the pathogenesis of this chronic disease associated with bacterial colonization and uncontrolled inflammation [25]. The used mouse model of CF shows a chronic inflammation of the respiratory tract and probably an increased requirement of tissue repair (1.5-fold increased *Toll-like receptor 4* and approximately 40-fold increased *Trefoil factor 1*). Both additional functions of DMBT1 in angiogenesis and epithelial differentiation are important components of tissue regeneration. De Lisle et al. used different CF mice (cystic fibrosis transmembrane conductance regulator knockout mice; cftr^m1Unc^) and described dilated lumina containing protein or mucus plugs in pancreatic acini and crypts of the small intestine. Upregulation of the DMBT1 mouse homologue muclin/CRP-ductin was observed after appearance of these morphological changes [26–28]. So, upregulation of DMBT1 in CF is observed in the respiratory and gastrointestinal tract, both organs with many glands and multiple contacts to different bacteria, viruses, and pathogens.

### DMBT1 and mucus

The viscous mucus is a main problem in patients with CF. DMBT1 was detected in the mucus using immunohistochemistry. Alveoli were filled with DMBT1-positive mucus and it has to be assumed that DMBT1 locally inactivates surfactant in CF as described earlier in infants with respiratory distress syndrome [11]. The cilia, a protective mucus layer and an airway surface liquid layer are the components of the mucociliary apparatus. All of those three parts are important and work together to remove inhaled particles from the lung [29]. It is known that CF patients have huge problems with mucus clearance and take strong efforts to eliminate the viscous mucus out of their respiratory tract. Normally, human ciliated respiratory epithelial cells are able to remove mucus with low viscosity, but this is aggravated in cystic fibrosis because of the high viscosity of the mucus. Submucosal glands contain both, mucous and serous cells. The serous cells secrete proteoglycans and various antimicrobial proteins [30]. Glands associated with respiratory airways show DMBT1 expression [10]. Our study results demonstrate that treatment with human recombinant DMBT1 decreases ciliary motility of human ciliated respiratory epithelial cells in vitro. This finding suggests that increased DMBT1 levels in patients with CF may impede the ciliary function and thus mucus removal. In conclusion, DMBT1 seems to be a potential biomarker for airway inflammation in CF and CF lung disease. Further studies that analyze longitudinal samples of patients with CF along with their clinical data or at least cross-sectional data of patients with predefined, different stages of CF lung disease are needed in order to establish DMBT1 as a biomarker to monitor progression of CF lung disease. Further research that correlates DMBT1 levels in respiratory fluids (e.g., sputum, nasal lavage) with disease activity is needed. The described findings are not specific to DMBT1, but also occur in a comparable way for other inflammatory markers (e.g., neutrophil elastase [31, 32], and Cathepsin S [33]).

### DMBT1 and macrophages

Macrophages are able to express DMBT1 and otherwise, DMBT1 stimulates the random migration (chemokinesis) of macrophages [9, 34]. DMBT1 is highly expressed in pulmonary macrophages of the demonstrated CF patient. Alveolar macrophages take part in innate immunity and in removal of cell debris and of mucus [35]. The chronic inflammation in CF lung tissue is characterized by significant cell infiltration of neutrophils, T cells, and macrophages [36]. Macrophages are increasingly recognized in the context of CF as a key player in the initiation, perpetuation, and resolution of CF lung inflammation [36–38]. DMBT1 may support macrophages in their functions.

### DMBT1 and ACC

DMBT1 concentrations were significantly reduced in lung epithelial cells following treatment with ACC. Thus, ACC might be a therapeutic option to dampen elevated respiratory DMBT1 levels in CF patients. The exact mechanism of DMBT1 protein reduction remains unclear, but it is already known that ACC inhibits the synthesis of mucins and different proinflammatory mediators after virus infections in A549 cells [39, 40]. Other possibilities include inductions of proteolysis or reduced transcription of the *DMBT1* gene. The ACC concentration of approximately 2.5 mg/ml (15 mM) used in the present study could also be safely administered to children [41–43] and can be achieved if NAC is administered as inhalation preparation [44]. On the contrary, DMBT1 has also been shown to have anti-inflammatory properties in a mouse model of allergic rhinitis, another respiratory disease with marked inflammation [45–47]. Intranasal administration of DMBT1 in mice sensitized with albumin had a protective effect by inhibiting eosinophil infiltration in the nasal mucosa and significantly decreasing levels of interleukin-4 and interleukin-5 in bronchoalveolar lavage [47]. Therefore, DMBT1 seems to be a modulatory protein with pro- and anti-inflammatory functions [23]. New therapies should attempt to reduce upregulated DMBT1 levels in CF to physiological concentrations. 

However, the therapeutic effect of ACC in CF remains ambiguous. On the one hand, ACC has also been shown to reduce other inflammatory markers apart from DMBT1 in the airways, e.g., neutrophil count and elastase activity [48]. Moreover, Blasi et al. reported good antibacterial properties and an ability to interfere with biofilm formation in in vitro studies [49]. On the other hand, the clinical impact of ACC therapy in patients with CF remains to be elucidated. A Cochrane review from 2013 showed no significant effect on primary clinical outcome parameters [50]. However, a placebo controlled trial by Conrad et al. detected a significant effect of ACC on FEV1 of CF patients, but not on the evaluated parameters of neutrophil inflammation [51]. In conclusion, a positive effect of ACC therapy (inhaled or oral) on clinical outcome/on airway inflammation cannot be ruled out, as studies that use more sensitive parameters such as multiple breath washout or MRI imaging are lacking in the literature. To ensure effective ACC therapy, the drug has to be administered directly into the airways. One conceivable possibility may be the use of ACC-containing fluid instilled during bronchoalveolar lavage to reduce DMBT1 levels in CF patients with high DMBT1 expression.

## Limitations

The limitations of this study include the restricted number of lung tissue from CF patients. We are not able to present a study collective representing disease progression or cross-sectional data with predefined, different stages of CF lung disease. The A549 cells used to elucidate the effect of ACC on DMBT1 expression represent a lung cancer cell line. Differences between this cancer cell line and commercially ordered lung epithelial cells have been described and have to be considered when interpreting the presented results. Additionally, the exact mechanism of DMBT1 reduction by ACC remains unclear and needs further research.

## Conclusions

In conclusion, DMBT1 expression is upregulated in lung tissue of CF patients and of a transgenic mouse model of CF-like lung disease and may be used as a potential biomarker to diagnose and monitor CF lung disease. Increased DMBT1 levels negatively influence cilia motility of human respiratory epithelial cells. Treatment of human lung epithelial cells with ACC leads to reduced DMBT1 concentrations, thus providing a new molecular mechanism of ACC function in CF.

## Supplementary Information

**Additional file 1.** Standard curve-DMBT1 ELISA.

**Additional file 2.** Ciliary motility without hrDMBT1.

**Additional file 3.** Ciliary motility with hrDMBT1.

## Data Availability

The datasets used and/or analyzed during the current study are available from the corresponding author on reasonable request.
