# Supplementary figures and images for: DMBT1 is upregulated in cystic fibrosis, affects ciliary motility, and is reduced by acetylcysteine
Source: Mol Cell Pediatr. 2022 Mar 5;9:4. doi: 10.1186/s40348-022-00136-0 (PMC8898207; doi:10.1186/s40348-022-00136-0)

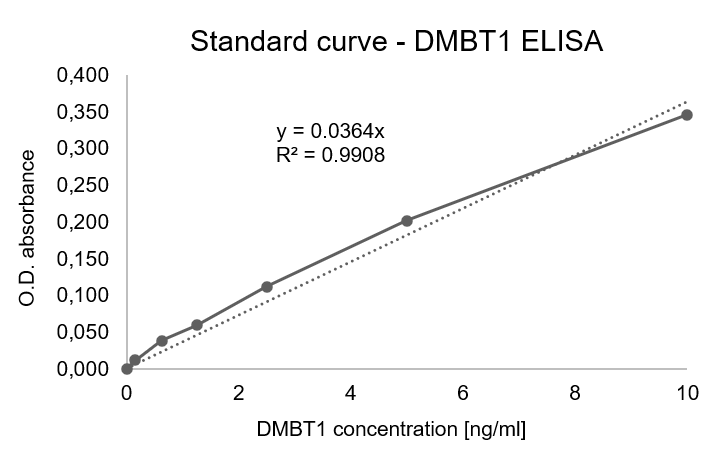

Supplement: Supplementary file 1 — Additional file 1. Standard curve-DMBT1 ELISA. [file 40348_2022_136_MOESM1_ESM.tif]
